# Correlation of pyroglutamate amyloid β and ptau Ser202/Thr205 levels in Alzheimer’s disease and related murine models

**DOI:** 10.1371/journal.pone.0235543

**Published:** 2020-07-09

**Authors:** Joerg Neddens, Magdalena Daurer, Stefanie Flunkert, Kerstin Beutl, Tina Loeffler, Lauren Walker, Johannes Attems, Birgit Hutter-Paier

**Affiliations:** 1 QPS Austria GmbH, Grambach, Austria; 2 FH Joanneum Graz, Graz, Austria; 3 Translational and Clinical Research Institute and Newcastle University Institute for Ageing, Campus for Ageing and Vitality, Newcastle upon Tyne, United Kingdom; Nathan S Kline Institute, UNITED STATES

## Abstract

Senile plaques frequently contain Aβ-pE(3), a N-terminally truncated Aβ species that is more closely linked to AD compared to other Aβ species. Tau protein is highly phosphorylated at several residues in AD, and specifically phosphorylation at Ser202/Thr205 is known to be increased in AD. Several studies suggest that formation of plaques and tau phosphorylation might be linked to each other. To evaluate if Aβ-pE(3) and ptau Ser202/Thr205 levels correlate in human and transgenic AD mouse models, we analyzed human cortical and hippocampal brain tissue of different Braak stages as well as murine brain tissue of two transgenic mouse models for levels of Aβ-pE(3) and ptau Ser202/Thr205 and correlated the data. Our results show that Aβ-pE(3) formation is increased at early Braak stages while ptau Ser202/Thr205 mostly increases at later stages. Further analyses revealed strongest correlations between the two pathologies in the temporal, frontal, cingulate, and occipital cortex, however correlation in the hippocampus was weaker. Evaluation of murine transgenic brain tissue demonstrated a slow but steady increase of Aβ-pE(3) from 6 to 12 months of age in the cortex and hippocampus of APP_SL_ mice, and a very early and strong Aβ-pE(3) increase in 5xFAD mice. ptau Ser202/Thr205 levels increased at the age of 9 months in APP_SL_ mice and at 6 months in 5xFAD mice. Our results show that Aβ-pE(3) and ptau Ser202/Thr205 levels strongly correlate in human as well as murine tissues, suggesting that tau phosphorylation might be amplified by Aβ-pE(3).

## Introduction

Alzheimer’s disease (AD) is characterized by two major pathologies, aggregated tau and amyloid β (Aβ) plaques.

Tau pathology manifests as neurofibrillary tangles and neuropil threads, and strongly depends on posttranslational modifications of tau. Phosphorylation at different residues has been evaluated in detail (for review see [1]). Phosphorylated tau (ptau) at Ser202/Thr205 is the most widely analyzed residue that can be labeled by the AT8 antibody. This antibody is routinely used by pathologists to determine the neurofibrillary tangle Braak stage in *post mortem* brains [2, 3].

Aβ plaques are composed of several different Aβ variants with N- and C-terminal modifications [4–6]. The most abundant variants in AD are Aβ_1–40_, Aβ_1–42_, pyroglutamate Aβ (Aβ-pE(3)) and Aβ_4–42_ [7–9]. While C-terminally modified Aβ variants have been the focus of AD research for several decades [10–12], N-terminally modified variants, such as Aβ-pE(3), are just recently gaining attention. Senile plaques that contain Aβ-pE(3) are frequent [13], and Aβ-pE(3) is more closely linked to AD [14] while Aβ_1-x_ is seen in 80% of individuals aged over 80 years irrespective of their cognitive status [15]. Aβ-pE(3) is shown to have a faster aggregation kinetic and is thus more prone to aggregation and formation of fibrils [16–22] due to increased hydrophobicity and altered pH-dependent solubility [19]. In murine primary cortical neurons, Aβ-pE(3) increases lipid peroxidation [23], and causes neuron loss, gliosis, as well as learning and memory deficits *in vivo* [21, 24, 25]. Furthermore, it could be shown that diffuse plaques in sporadic AD cases have increased pyroglutamated Aβx-42 compared to cognitively unaffected amyloid positive individuals [26]. Processes involved in Aβ-pE(3) pathophysiology might thus play an early and critical role for the development of AD [13, 25]. Nussbaum and colleagues have shown that tau is required for Aβ-pE(3) cytotoxicity, fueling the importance of tau for AD pathology [25]. To validate the significance of tau for Aβ-pE(3) pathophysiology, Mandler and colleagues analyzed Aβ-pE(3), non-pE(3) Aβ and ptau Ser202/Thr205 levels in a large cohort of AD cases [27]. Their results suggest that Aβ-pE(3) formation can predict ptau Ser202/Thr205 load in some cortical brain regions while non-pE(3) Aβ failed to do so, supporting the hypothesis that Aβ-pE(3) represents a key link between Aβ and ptau [27]. The monoclonal Aβ-pE(3) antibody used in their study was previously generated and shown to be specific on human and mouse brain tissue [28]. To build on these findings, we measured Aβ-pE(3) and ptau in numerous brain regions of human and mouse tissue. This is of importance as the deposition of ptau and Aβ-pE(3) across the brain in AD follows distinct topographical patterns of distribution, with some areas affected early in the disease and others only in later stages. Results of this study may support the hypothesis that Aβ-pE(3) plays a central role in the early development of AD. We thus quantified and correlated Aβ-pE(3) and ptau Ser202/Thr205 by labeling with AT8 antibody in human tissue of different Braak stages as well as in two AD mouse models over age. To ensure antibody specificity on human and mouse tissue, two Aβ-pE(3) antibodies of different origin were compared for binding capacities on both tissue types before start of the main study.

## Material and methods

### Human brain samples

Paraffin sections of 6 μm thickness from human *post mortem* brains showing Braak stages 0, I/II, III/IV and V/VI [3] were provided by the Newcastle Brain Tissue Resource (NBTR), Newcastle University, UK, in accordance with the approval of the joint Ethics Committee of Newcastle and north Tyneside Health Authority and following NBTR brain banking procedures. Briefly, brains were processed as follows; at autopsy, the right hemisphere, cerebellum, and brainstem were immersion fixed in 4% formalin. Following fixation the right hemisphere was sub-dissected, dehydrated, and processed into paraffin wax. The neuropathological diagnosis was performed according to internationally accepted criteria (Table 1) [29]. Here, we investigated 5 neuroanatomical regions, which partly reflect the topographical spreading of tau pathology according to Braak stages: hippocampus (HC), temporal cortex (TeCtx), cingulate cortex (CiCtx), occipital cortex (OcCtx), and frontal cortex (FrCtx).

**Table 1:** Individual human case information.

	Case	Age	Sex	*Post mortem* dely to fixation in hours	Fixation time in weeks	Cognitive status	Braak stage	Thal phase	CERAD score	NIA-AA score
**Braak 0**	1	68	M	54	7	Cognitively normal	0	0	Negative	Not
	2	55	M	41	11	Cognitively normal	0	0	Negative	Not
	3	70	M	72	6	Cognitively normal	0	1	Negative	Not
	4	78	F	34	8	Cognitively normal	0	1	Negative	Low
	5	73	M	25	9	Cognitively normal	0	0	Negative	Not
**Braak I/II**	6	96	F	114	49	Mild dementia	2	3	Negative	Low
	7	77	M	83	15	Cognitively normal	2	3	Negative	Low
	8	94	F	15	9	Cognitively normal	2	1	Negative	Low
	9	70	M	39	7	Multiple psychiatric and physical problems	1	1	Negative	Low
	10	74	F	49	10	Cognitively normal	1	0	Negative	Not
**Braak III/IV**	11	75	M	82	23	Cognitively normal	4	3	Moderate	Intermediate
	12	79	M	13	15	Cognitively normal	3	4	Frequent	Intermediate
	13	81	M	82	8	Unspecified dementia	3	2	Sparse	Low
	14	98	F	59	8	Cognitively normal	3	3	Sparse	Intermediate
	15	91	M	48	9	Moderate cognitive impairment and vascular disease	3	4	Sparse	Intermediate
**Braak V/VI**	16	84	F	47	16	Severe dementia, anxiety and depression	6	5	Frequent	High
	17	77	F	63	5	Dementia	6	5	Frequent	High
	18	80	F	32	16	Dementia	6	5	Frequent	High
	19	86	F	5	6	Dementia	6	5	Frequent	High
	20	89	F	85	8	Dementia	6	5	Frequent	High

Table 1 gives detailed information about human cases showing age, sex, fixation details, cognitive status, Thal phases of β-amyloid, CERAD score for neuritic plaques, and level of AD neuropathologic changes according to NIA-AA guidelines.

#### Murine brain samples

Brain tissue from female 6, 9 and 12 months old APP_SL_ transgenic, and 5xFAD transgenic and non-transgenic littermates was used. APP_SL_ transgenic mice overexpress human amyloid precursor protein (APP)751 with Swedish (APP670/671) and London (717) mutations under the regulatory control of the Thy-1 promoter [30–32]. 5xFAD (familial AD) transgenic mice overexpress human APP695 with Swedish (670/671), Florida (716) and London (717) mutation as well as human presenilin 1 with two mutations (146; 286) [33]. All animals were euthanized at the above mentioned age with a pentobarbital overdose and flush-perfused transcardially with 0.9% saline through the left ventricle. Brains were dissected and hemispheres immersion-fixed in 4% paraformaldehyde in 0.1 M phosphate buffer, pH 7.4, for 2 h at room temperature (RT). Afterwards, tissue was cryoprotected in 15% sucrose in phosphate buffer saline (PBS) overnight at 4°C, and then embedded in tissue freezing medium (Leica Biosystems, Germany) in cryomolds and snap-frozen on dry ice-cooled liquid isopentane. Frozen samples were stored at -80°C until sectioning. Sagittal sections of 10 μm thickness were produced on a Leica CM1950 cryotome. Sections were mounted on polylysine slides (Thermo Scientific) and stored at -20°C. A total of 4–5 sections per animal from 4–5 different mediolateral levels throughout the whole hemisphere were used for immunofluorescent labeling.

### Ethics approval and consent to participate

Human tissue was provided by the Newcastle Brain Tissue Resource (NBTR), Newcastle University, UK in accordance with the approval of the joint Ethics Committee of Newcastle and North Tyneside Health Authority and following NBTR brain banking procedures.

All experiments Including animal tissue were performed in accordance with the Austrian guidelines for the care and use of laboratory animals (Tierversuchsgesetz 2012-TVG 2012,BGBl. I Nr. 114/2012). Animal housing and euthanasia were approved by the Styrian government (Amt der Steiermärkischen Landesregierung, Abteilung 13 –Umwelt und Raumordnung, Graz, Austria; ABT13-78Jo115/2013-2016; ABT13-78Jo118/2013-13).

### Immunofluorescent labeling

Human brain sections were deparaffinized for 10 min in Tissue Clear (Sakura, 1466, Netherlands) and 5 min in Tissue Clear/100% ethanol, washed for 5 min in 100% ethanol, and then subsequently rehydrated with decreasing alcohol concentrations (96%, 70% and 50% ethanol for 2 min each). Thereafter, sections were treated with 1x citrate buffer (Thermo Scientific, AP-9003, California, USA) at 95°C in a steamer for antigen retrieval and cooled down to RT for another 15 min.

Frozen mouse brain sections were air-dried and then washed in PBS for 10 min.

Additionally, sections (except for combined visualization of rbAβ-pE(3), ptau Ser202/Thr205 and cell nuclei) were pretreated with ice-cold sodium borohydride/PBS solution (1 mg/ml; Sigma-Aldrich, 213462) for 4 min and washed in PBS twice for 5 min each. Sections labeled with Thioflavin S (ThioS) were then stained with a 0.5% ThioS solution for 7 min and again washed twice in PBS.

Following this, non-specific labeling of human and mouse brain sections was blocked by incubating sections for 60 min with 10% donkey serum/0.3% Triton X-100/PBS or 10% M.O.M Blocking Reagent/0.3% Triton X-100/PBS. For antigen detection, sections were incubated with primary antibodies (see Table 2) in a damp chamber over night at 4°C. After washing, primary antibody binding was visualized by incubating sections with secondary fluorophore conjugated antibodies (Table 3) for 60 min. Cell nuclei were visualized by counterstaining with 4′,6-Diamidin-2-phenylindol-working solution (DAPI, AppliChem, A1001) for 15 min and subsequently washed in PBS and ddH_2_O for 5 min each. Sections were covered with Moviol and coverslips.

**Table 2:** List of primary antibodies.

Antigen	Clone	Species	Source	Order #	Dilution	Antibody ID
Aβ (aa 1–16)	6E10	mouse	Biolegend, San Diego, USA	803003	1:1000	AB_2564652
msAβ-pE(3)	K17	mouse	Vivoryon AG, Halle, Germany	N/A	1:2000	N/A
NeuN (aa 1–97)	poly	guinea pig	Synaptic Systems GmbH, Goettingen, Germany	266 004	1:2000	AB_2619988
rbAβ-pE(3)	poly	rabbit	Synaptic Systems GmbH, Goettingen, Germany	218 003	1:500 (mouse: quantification experiment); 1:2000 (human; mouse: antibody evaluation experiments)	AB_2056424
pSer202/Thr205 tau	AT8	mouse	Thermo Scientific, Illinois, USA	MN1020	1:300 (mouse); 1:100 (human)	AB_223647
Glial Fibrillary Acidic Protein (GFAP)	poly	goat	Abcam, Cambridge, UK	ab53554	1:1000	AB_880202

**Table 3:** List of secondary antibodies.

Antibody	Conjugation	Source	Order #	To visualize	Antibody ID
Donkey Anti-Mouse IgG H&L	AlexaFluor 555	Abcam, Cambridge, UK	ab150110	Aβ (aa 1–16)	AB_2783637
Donkey Anti-Guinea Pig IgG H&L	Alexa Fluor 488	Jackson ImmunoResearch Laboratories, Inc., Pennsylvania, USA	706-545-148	NeuN (aa 1–97)	AB_2340472
Donkey Anti-Rabbit IgG H&L	DyLight 650	Abcam, Cambridge, UK	ab96922	rbAβ-pE(3) (quantification experiment)	AB_10680408
Donkey Anti-Rabbit IgG H&L	AlexaFluor 555	Abcam, Cambridge, UK	ab150066	rbAβ-pE(3)	N/A
Donkey Anti-Mouse IgG H&L	DyLight 650	Abcam, Cambridge, UK	ab98797	- pSer202/Thr205 tau - ms rbAβ-pE(3)	AB_10674087
Donkey Anti-Goat IgG H&L	DyLight 550	Abcam, Cambridge, UK	ab96936	GFAP	AB_10679832
**Dilution of secondary antibodies was 1:500**					

### Imaging and image analysis

Imaging of immunofluorescent labeling was performed using a Zeiss AxioImager.Z1 microscope with a high aperture lens and an AxioVision 4.8 software-driven AxioCam MRm digital camera (10x lens, numeric aperture 0.8, 1x optocoupler).

Mosaic image arrays covering all cortical layers and underlying white matter of human labeled brain sections (covering a mean size of 18 mm^2^) were captured at different z-levels and projected to 2D using the AxioVision 4.8 software. On sections of human neocortical areas, two of these image arrays were recorded per target area, whereas one array was recorded from the hippocampal formation covering parts of the subiculum, dentate gyrus, and CA3. In contrast, the whole isocortex and hippocampus were recorded on mouse brain sections.

Quantitative image analysis was performed using Image-Pro Plus (version 6.2, Media Cybernetics, Inc., Rockville, USA). Grey-scale single channel images were corrected for background intensities using lowpass filtering, and signal from autofluorescent objects (mostly lipofuscin and erythrocytes) was subtracted from the channel used for immunofluorescent labeling. Objects were identified by a combination of Edge+ filter, adequate thresholding, and size and shape restrictions. After defining the parameters for detecting the targeted objects, macro-driven quantitative image analysis was performed automatically using the same parameters on all images to evaluate the immunoreactive area (IR) in percent. The quantitative results are therefore unbiased, rater-independent and fully reproducible.

### Western blotting

25 and 50 ng of peptide of Aβ-pE(3–40), Aβ-pE(3–42), Aβ 4–42 and Aβ 1–42 (AnaSpec, Fremont, CA, USA, #AS-29906; #AS-29907; #AS-29908 and rPeptide, Watkinsville, GA 30677, USA, #A1167-2) were separated by molecular weight on a 18% SDS-PAGE polyacrylamide gel. A protein marker visualized correct separation of the peptides and confirmed the correct peptide band size. Subsequently, peptides were transferred onto a 0.45 μm nitrocellulose membrane using a semi-dry blot chamber (Bio-Rad, Hercules, USA) and membranes were blocked with 5% non-fat dry milk in 1 x TBS for 1 h. Primary antibodies msAβ-pE(3) rbAβ-pE(3) were used in a concentration of 1:1000 and incubated for 2 h at room temperature. Afterwards, membranes were washed in TBS and incubated in horseradish peroxidase-coupled secondary antibodies for 1 h at room temperature (1:5000; donkey-anti-rabbit IgG: NA934/ GENA934; sheep-anti-mouse IgG: NXA931; GE-Healthcare, Little Chalfont, UK). Wester-Bright ECL spray (Advansta, Menlo Park, USA) and C-digit blot scanner (Licor) were used for the visualization.

### Statistical analyses

All statistical analyses and preparation of graphs were conducted using Graph-Pad Prism (version 4.03, San Diego, CA, USA). Descriptive statistical analyses were performed on all evaluated parameters including the evaluation of normal distribution using the Kolmogorov-Smirnov test.

Group variances were calculated either by One-way or Two-way ANOVA. If a significant interaction among groups was detected, Newman-Keuls or Bonferroni’s *post hoc* analysis was performed. Correlation analyses were performed using Pearson or Spearman correlation depending on normal distribution analyzed by Kolmogorov-Smirnov test. A detailed description of performed statistical analyses is given in the corresponding figure legend. Data were averaged and are presented as mean + standard error of mean (SEM). An α-error level of p<0.05 was considered significant.

## Results

### Comparison of the specificity of two Aβ-pE(3) antibodies in diseased human and mouse tissue

**Fig 1:**
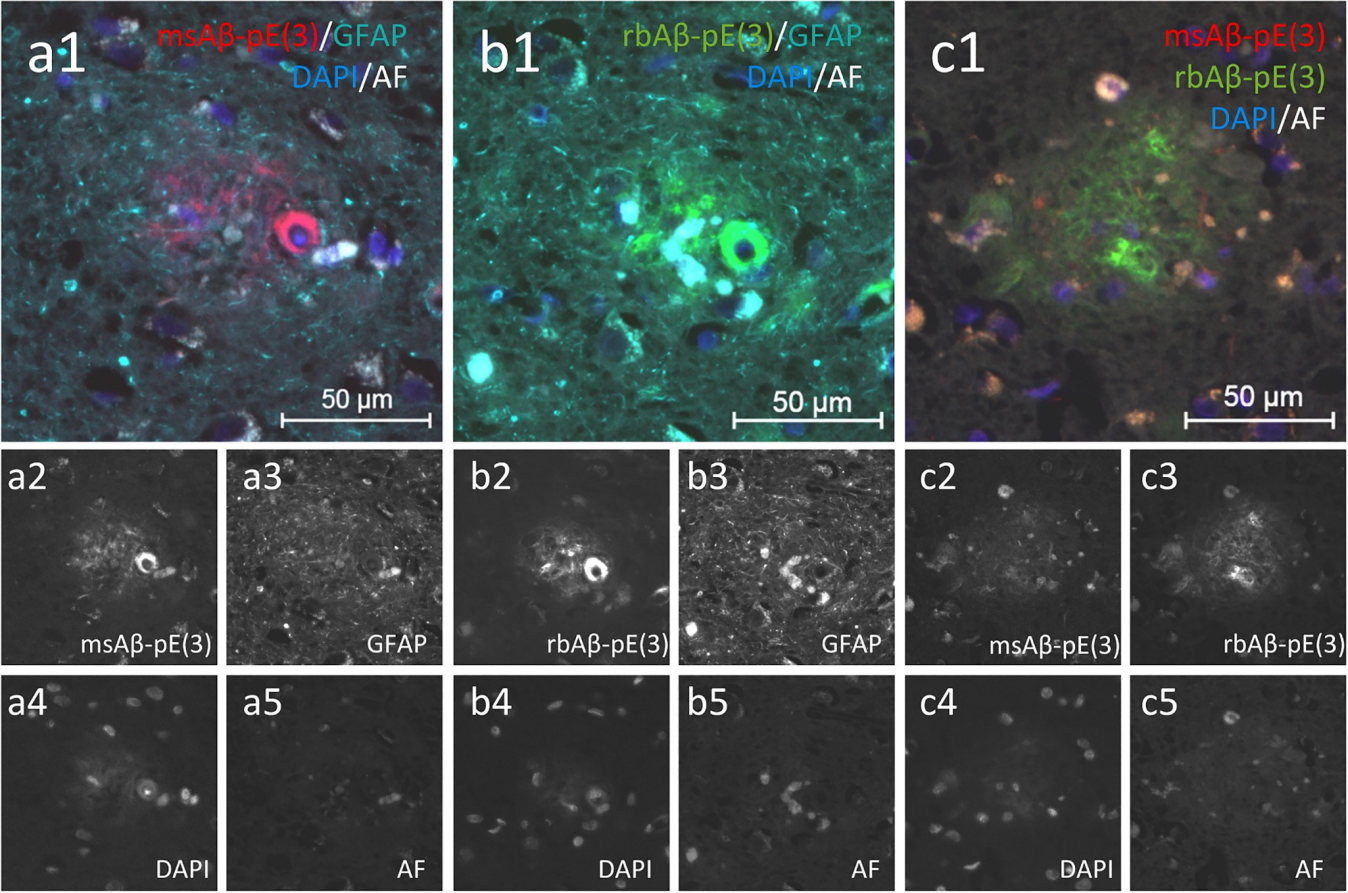
Immunofluorescent labeling of human cortical tissue of Braak stage V/VI with two different antibodies recognizing Aβ-pE(3), GFAP, and labeling of cell nuclei with DAPI. Separate labeling of Aβ-pE(3) with antibodies raised in either mouse (msAβ-pE(3) **a1, a2**) or rabbit (rbAβ-pE(3) **b1, b2**) resulted in a strong and similar immunoreactive area. Both antibodies were additionally co-labeled with an antibody against GFAP (**a1, a3, b1, b3**) and cell nuclei were stained with DAPI (**a1-c1, a4-c4**). Double labeling with both Aβ-pE(3) antibodies (**c1-c3**) revealed no major differences in the immunoreactive area of the rbAβ-pE(3) (**c3**) compared to msAβ-pE(3) (**c2**) antibody. An imaging control of autofluorescence was recorded to verify that epifluorescent signal comes from immunoreactivity of Aβ-pE(3) and GFAP labeling (**a5-c5**). AF: autofluorescence, Aβ-pE(3): pyroglutamate Aβ, DAPI: 4′,6-Diamidin-2-phenylindol, GFAP: glial fibrillary acidic protein. Pictures were taken from a Braak stage V/VI case (case 18).

To evaluate the specificity of two Aβ-pE(3) antibodies, different labeling was performed in human frontal cortical tissue of cases that were Braak stage V/VI, as well as cortical and hippocampal tissue of AD mouse models. Immunofluorescent labeling of human cortical tissue of Braak stage V/VI cases showed a strong Aβ-pE(3) signal after labeling with Aβ-pE(3) antibodies of mouse (msAβ-pE(3); Fig 1a1), as well as rabbit (rbAβ-pE(3); Fig 1b1) origin. Both antibodies were used for co-labeling with a GFAP specific antibody and DAPI, identifying astrocytosis and cell nuclei, respectively (Fig 1a1–1a4 and 1b1–1b4). GFAP labeling was performed to confirm uniform labeling between slices so differences between Aβ-pE(3) antibodies are comparable. Co-labeling of human tissues for Aβ-pE(3) with the mouse and rabbit antibodies revealed no major differences in the labeling pattern of these two antibodies (Fig 1c1–1c3). During antibody tests, human tissue was also labeled with the same antibody combination but without antigen retrieval resulting in a less intense labeling (S1 Fig).

To analyze if the two Aβ-pE(3) antibodies have a comparable binding specificity on murine tissue as observed on human tissue, brain tissue of APP_SL_ and 5xFAD mice was labeled accordingly (S2 Fig). In both animal models, a strong overlap of msAβ-pE(3) and rbAβ-pE(3) signal could be observed (S2a and S2b Fig). Double labeling of plaque associated Aβ-pE(3) in the cortex of APP_SL_ mice using the rbAβ-pE(3) and msAβ-pE(3) antibody revealed no differences in the labeling pattern of the rbAβ-pE(3) antibody compared to the msAβ-pE(3) antibody in diffuse (S2g1–S2g3 Fig) as well as dense core plaques (S2h1–S2h3 Fig). A similar effect could be observed in 5xFAD mice (S2i1–S2i3 and S2k1–S2k3 Fig). Labeling of the hippocampus of a 12 month old non-transgenic animal revealed a signal absence for the msAβ-pE(3) and rbAβ-pE(3) antibody but an adequate signal of GFAP positive astrocytes validating the specificity of both antibodies(S3 Fig).

To further validate the specificity of the rbAβ-pE(3) antibody, co-labeling with the 6E10 antibody and Thioflavin S (ThioS) staining was performed on brain tissue of APP_SL_ (S4a Fig) and 5xFAD (S4b Fig) mice. In both animal models, 6E10 labeling as specific anti-β-amyloid antibody staining, resulted in the strongest signal, and the rbAβ-pE(3) signal partly overlapped with the 6E10 signal, thus validating that Aβ-pE(3) is only a part of total Aβ load. ThioS labeled only the cored plaques and partially also overlapped with the 6E10 and rbAβ-pE(3) signal, although it is additionally supposed to label neurofibrillary tangles (S4 Fig).

In a final validation step, the specificity of both antibodies to Aβ-pE(3) was evaluated by Western Blot (S5 Fig). Our results show that both antibodies, msAβ-pE(3) and rbAβ-pE(3), are specifically labeling Aβ-pE(3–42). Both antibodies were also tested to label Aβ-pE(3–40), Aβ 4–42 and Aβ 1–42 but Western blotting resulted in no signal at all (S5 Fig).

Since the obtained results of the two tested antibodies were almost interchangeable, the rbAβ-pE(3) antibody was used for all further quantifications as it is commercially available, allowing other researchers to easily repeat experiments.

### Quantification of Aβ-pE(3) and ptau Ser202/Thr205 in human tissue of different Braak stages

**Fig 2:**
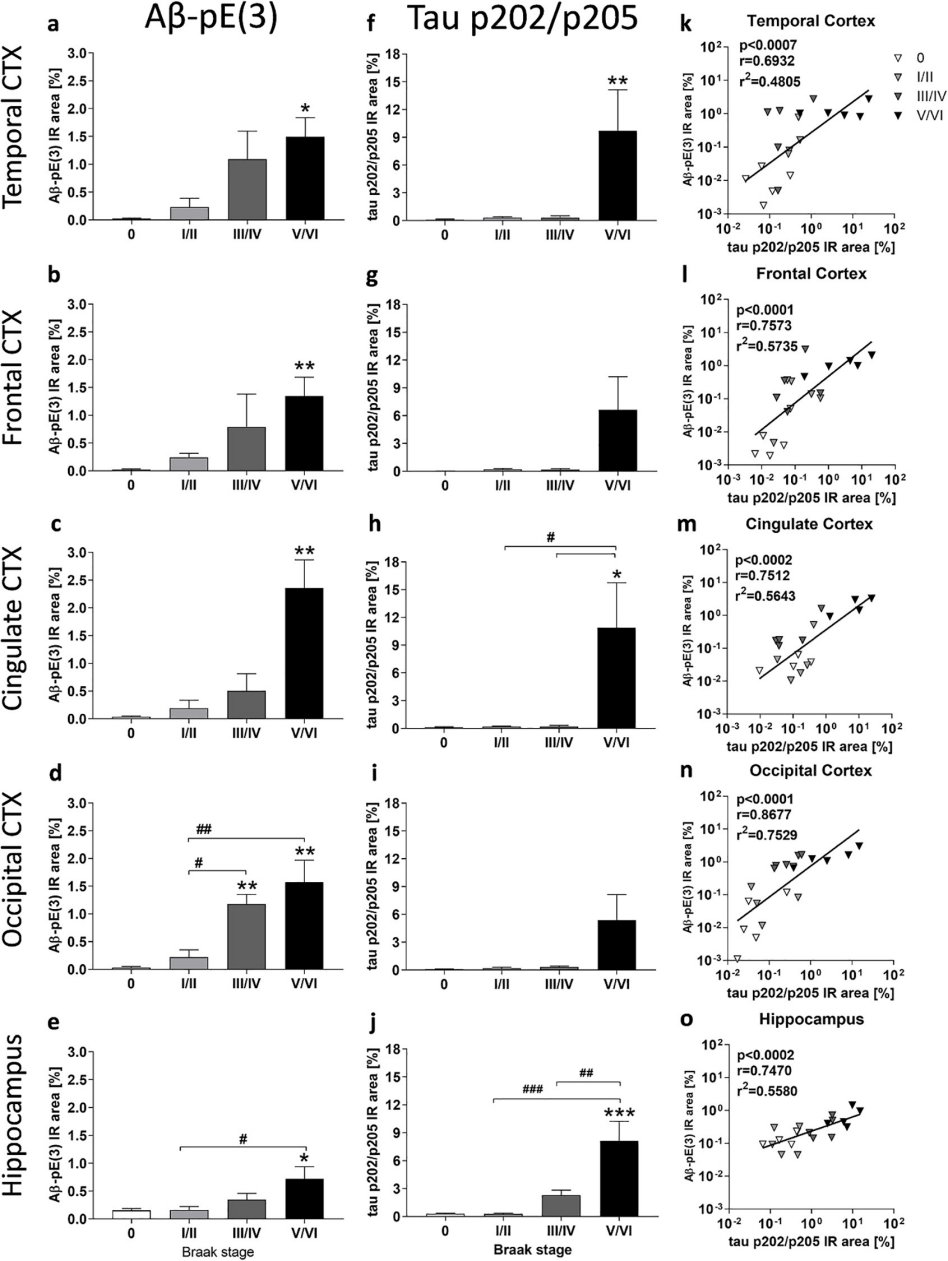
Quantification and correlation of Aβ-pE(3) and phosphorylated tau at Ser202/Thr205 in the cortex and hippocampus of AD and control cases. Aβ-pE(3) (**a-e**) and ptau Ser202/Thr205 (**f-j**) immunoreactive area and correlation of both quantifications (**k-o**) in the temporal (**a, f, k**), frontal (**b, g, l**), cingulate (**c, h, m**) and occipital (**d, i, n**) cortex as well as hippocampus (**e, j, o**) of human samples of Braak stages 0-VI. **a-c, f, g, i**: Kruskal-Wallis test with Dunn’s multiple comparison test; **d, e, h, j**: One-way ANOVA with Bonferroni’s multiple comparison test; Mean + SEM; n = 5 (except cingulated cortex, stage V/VI: n = 4), *comparison to Braak stage 0 or as indicated; ^#^differences between Braak stages; *p<0.05; **p<0.01; ***p<0.001. Spearman (**k, n**) and Pearson (**l, m, o**) correlation. Shown are the p-, r- and r^2^- values over all Braak stages including Braak stage 0. IR: immunoreactive area.

In order to evaluate Aβ-pE(3) levels and phosphorylation levels of tau at residue Ser202/Thr205, human brain tissue was labeled with the rbAβ-pE(3) and AT8 antibody, respectively. Quantification of the rbAβ-pE(3) immunoreactive area (IR) in isocortical regions of the human brain of different Braak stages, revealed a progressive increase of Aβ-pE(3) IR area with increasing Braak staging in the temporal, frontal, cingulate and occipital cortex as well as the hippocampus (Fig 2a–2e). In all areas the increase was significant at Braak stage V/VI compared to Braak stage 0 (temporal: p = 0.0277; frontal: p = 0.0025; cingulate: p = 0.0061; occipital: p = 0.0011). In the occipital cortex a significant increase in the IR area was additionally observed at Braak stage III/IV compared to Braak stage 0 (p = 0.0098) and also at Braak stage III/IV and V/VI compared to Braak stage I/II (p = 0.0251; p = 0.0031, respectively) suggesting a significant signal increase (Fig 2d). Also in the hippocampus an increase of rbAβ-pE(3) IR area between Braak stage I/II and III/IV (p = 0.0326) could be observed (Fig 2e).

Quantification of the AT8 immunoreactive area (IR) in isocortical regions of the human brain of different Braak stages revealed an increase of ptau Ser202/Thr205 at Braak stage V/VI in the temporal (p = 0.0055), frontal (p > 0.05), cingulate (p = 0.0233) and occipital cortex (p = 0.0031) compared to Braak stage 0 (Fig 2f–2i). In the hippocampus an increase in the ptau Ser202/Thr205 signal could be observed in Braak stage V/VI compared to Braak stage 0 (p = 0.0005), I/II (p = 0.0005), and III/IV (p = 0.0074; Fig 2j).

### Correlation analyses of results in human tissue of different Braak stages

Correlation analyses between Aβ-pE(3) and ptau Ser202/Thr205 levels were performed using Pearson or Spearman correlation depending on normal distribution (Fig 2k–2o). The analysis revealed a highly significant correlation between the parameters in the temporal, frontal, cingulate and occipital cortex as well as hippocampus (Fig 2k–2o). The coefficient of determination, r^2^, was highest in the occipital cortex (r^2^ = 0.7529; Fig 2n) followed by the frontal cortex (r^2^ = 0.5735; Fig 2l).

### Quantification of Aβ-pE(3) and ptau Ser202/Thr205 in two murine AD models

**Fig 3:**
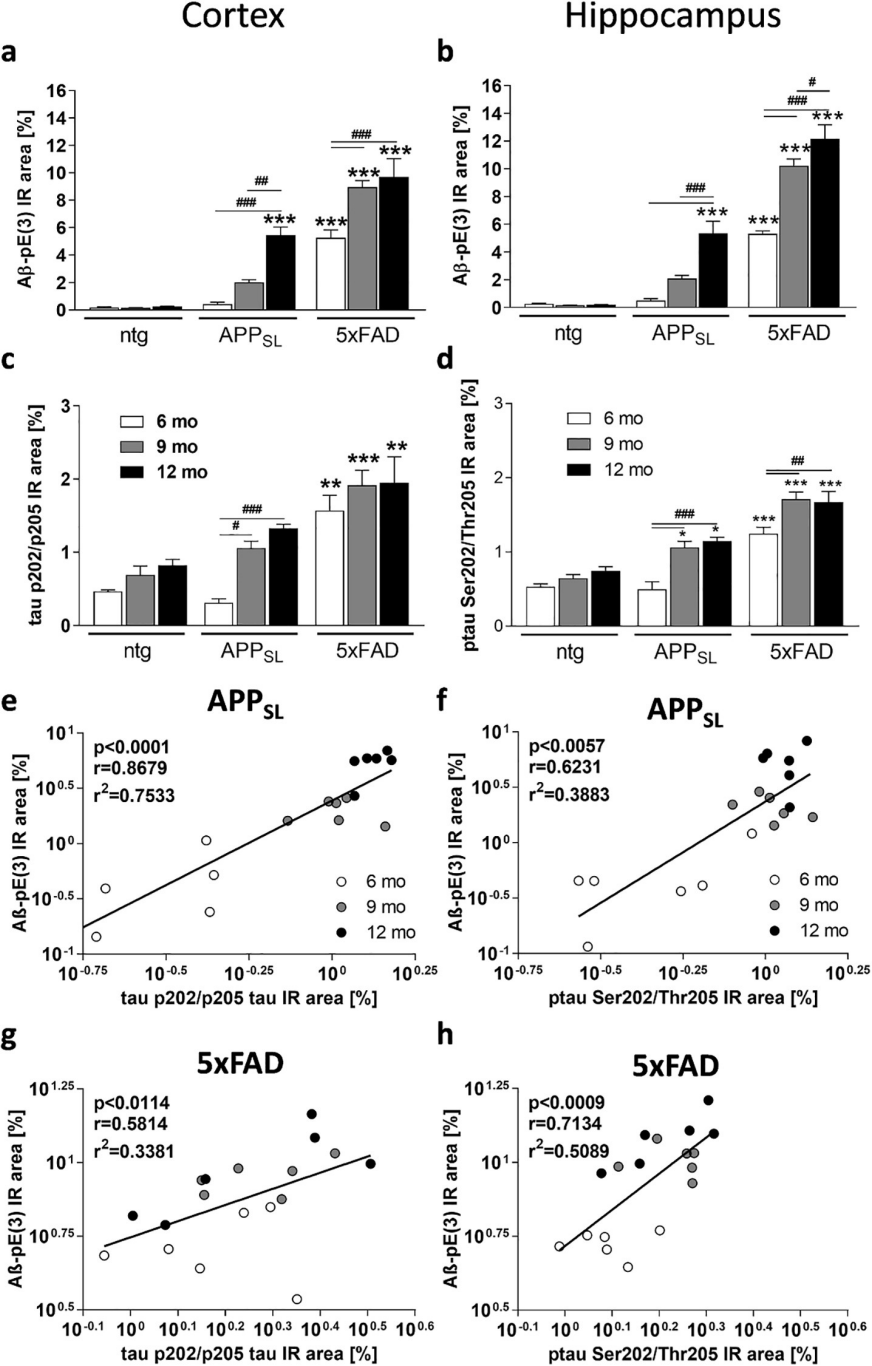
Quantification and correlation of Aβ-pE(3) and phosphorylated tau at Ser202/Thr205 in the cortex and hippocampus of murine AD models. Aβ-pE(3) (**a, b**) and ptau Ser202/Thr205 (**c, d**) immunoreactive area and correlation of both quantifications (**e-h**) in the cortex (**a, c, e, g**) and hippocampus (**b, d, f, h**) of 6, 9 and 12 month old APP_SL_ and 5xFAD transgenic as well as non-transgenic mice. **a-d**: Two-way ANOVA with Bonferroni’s *post hoc* test. Mean + SEM; ntg: n = 3, tg: n = 6. *comparison to ntg or as indicated; ^#^differences between age groups of one genotype; *p<0.05; **p<0.01; ***p<0.001. Spearman (**e**) and Pearson (**f-h**) correlation. Shown are the p-, r- and r^2^- values over all age groups including ntg littermates. IR: immunoreactive area, ntg: non-transgenic.

In order to evaluate Aβ-pE(3) levels and phosphorylation levels of tau at residue Ser202/Thr205, brain tissue of APP_SL_ and 5xFAD mice was labeled with the rbAβ-pE(3) and AT8 antibody, respectively. Analysis of Aβ-pE(3) levels in the cortex and hippocampus of APP_SL_ and 5xFAD mice showed in both models a significant signal increase with increasing age (Fig 3a and 3b). While only 12 month old APP_SL_ transgenic mice showed a significant increase in the rbAβ-pE(3) IR area in the cortex and hippocampus compared to non-transgenic littermates, 5xFAD mice demonstrated a significantly increased rbAβ-pE(3) IR area at 6 months that further increased with age. Statistical analysis of the progression revealed highly significant results in both mouse models that were strongest in the hippocampus of 5xFAD mice. It is worth mentioning, that the levels of Aβ-pE(3) in 5xFAD were much higher than in APP_SL_ mice, reaching comparable levels at 6 months of age to those observed in in APP_SL_ mice at 12 months (Fig 3a and 3b).

Analysis of ptau Ser202/Thr205 IR area in the cortex and hippocampus of 6 to 12 month old APP_SL_ and 5xFAD mice showed in both models a significant signal increase with age, although both models express murine tau only (Fig 3c and 3d). Also the non-transgenic littermates showed a slight increase in the ptau Ser202/Thr205 signal with increasing age, however the differences were not significant. Although a significant progression of ptau Ser202/Thr205 signal could be observed in the cortex of APP_SL_ mice, comparing each age group with age matched non-transgenic littermates resulted in no significant differences (Fig 3c). The ptau Ser202/Thr205 signal in the cortex of 5xFAD mice was in all age groups significantly increased compared to non-transgenic littermates. Additionally, in the cortex of 5xFAD mice the signal seemed to plateau at 9 months, thus not further increasing over age (Fig 3c). In the hippocampus of APP_SL_ and 5xFAD mice ptau Ser202/Thr205 signal was significantly increased at the age of 9 and 12 months compared to non-transgenic littermates of the same age. Additionally, in the hippocampus of 5xFAD mice the signal also seemed to plateau already at 9 months of age (Fig 3d).

### Correlation analyses of results in murine AD models

Correlation analyses of Aβ-pE(3) and ptau Ser202/Thr205 levels in the cortex and hippocampus of APP_SL_ and 5xFAD mice revealed a highly significant correlation of the parameters in the cortex of APP_SL_ mice and the hippocampus of 5xFAD mice (Fig 3e and 3h) while the significance was slightly lower in the hippocampus of APP_SL_ mice and the cortex of 5xFAD mice (Fig 3f and 3g). The corresponding r^2^ coefficient of determination was thus highest in the cortex of APP_SL_ mice (r^2^ = 0.7533; Fig 3e) and lowest in the cortex of 5xFAD mice (r^2^ = 0.3381; Fig 3g).

**Fig 4:**
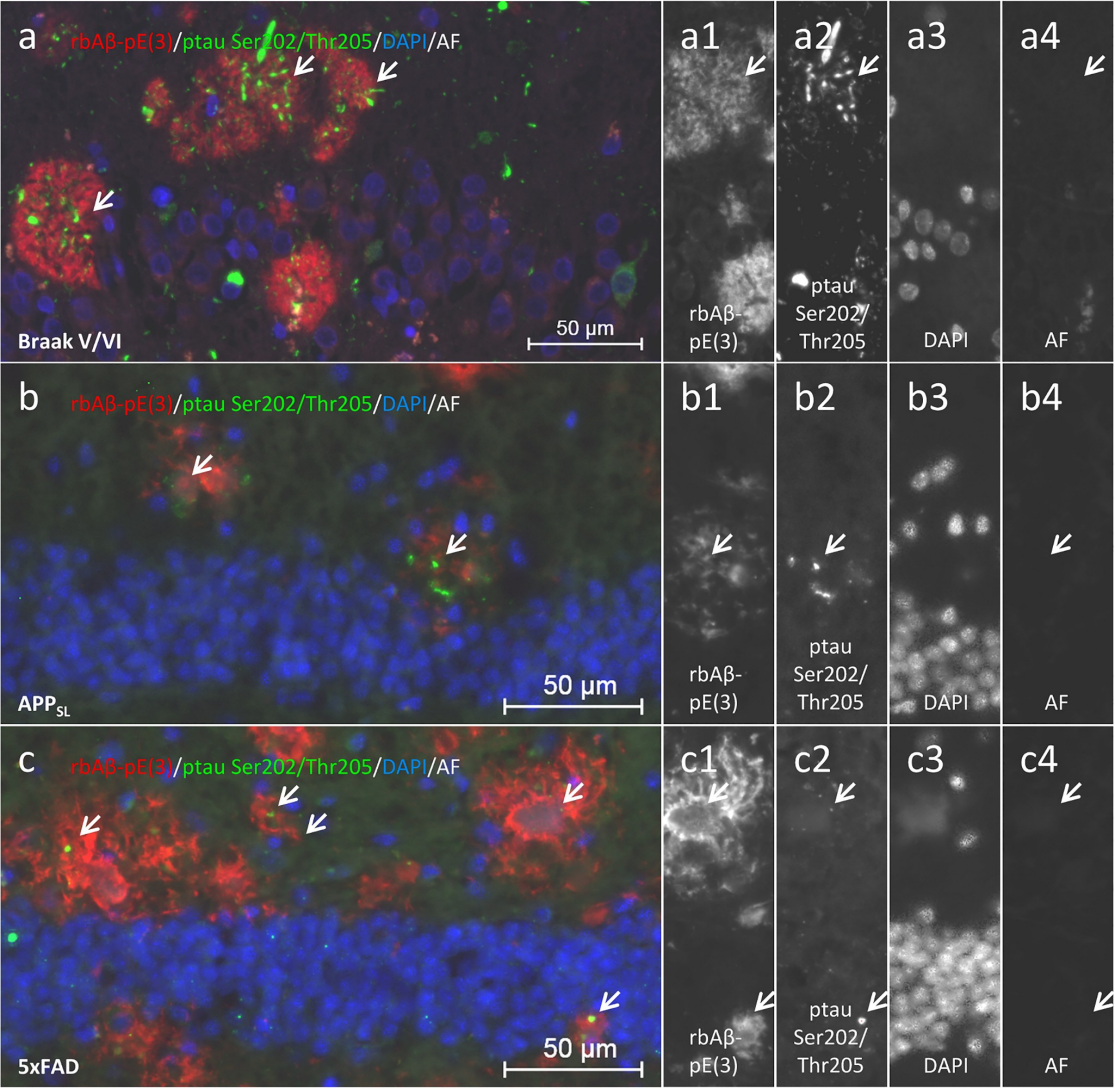
Double immunofluorescent labeling of Aβ-pE(3) and phosphorylated tau in the hippocampus of an AD subject (Braak stage V/VI) as well as 9 month old APP_SL_ and 5xFAD mice. Phosphorylation of tau at Ser202/Thr205 (AT8; **a-c**, **a2-c2**) occurs in human and mouse brain tissue close to accumulations of Aβ-pE(3) (**a-c**, **a1-c1**). All observed signals are not evident in the additionally recorded autofluorescence (AF) channel (**a-c**, **a4-c4**) as highlighted by white arrows. **a-c**, **a3-c3** cell nuclei visualized with DAPI. Pictures were taken from Braak stage V/VI (case 20).

Representative images of Aβ-pE(3) and ptau Ser202/Thr205 double labeling in human hippocampal tissue of Braak stage V/VI as well as hippocampal tissue of APP_SL_ and 5xFAD mice is shown in Fig 4.

In summary, our results show progressively increasing Aβ-pE(3) in the temporal, frontal, cingulate and occipital cortex as well as in the hippocampus of brain tissue of different Braak stages. Analysis of tau phosphorylation at Ser202/Thr205 revealed a sudden signal increase at Braak stage V/VI in all cortical regions and a progressive increase in the hippocampus of human tissue, resulting in the strongest correlation of Aβ-pE(3) and ptau Ser202/Thr205 in the occipital cortex. In the cortex and hippocampus of APP_SL_ transgenic mice both Aβ-pE(3) formation and ptau Ser202/Thr205 signal increased progressively, and was thus comparable to human tissue. Compared to APP_SL_ mice, progression of Aβ-pE(3) and ptau Ser202/Thr205 in the cortex and hippocampus of 5xFAD mice occured earlier, resulting in higher levels in older animals. Correlation analyses showed the strongest correlation in the cortex of APP_SL_ mice and the hippocampus of 5xFAD mice.

## Discussion

### Aβ-pE(3) in human tissue

Quantification of Aβ-pE(3) immunoreactive area in several cortical regions revealed a progressive increase of Aβ-pE(3) with increasing Braak stage. Specifically in the occipital cortex, Aβ-pE(3) levels were already significantly increased at Braak stage III/IV compared to Braak stage 0. Although the number of subjects analyzed in the presented study is relatively low, our results strengthen results by Mandler and colleagues while using a different antibody and additionally providing a more detailed evaluation of Aβ-pE(3) formation over Braak stages in several different cortical regions, thus providing a more in-depth analyses [27]. Previous studies did show that Aβ-pE(3) formation in AD cases and transgenic mice is closely associated with the localization and abundance of [11C]PIB autoradiographic signal, suggesting that Aβ-pE(3) is detectable by [11C]PIB, making Aβ-pE(3) a valuable target for the development of new therapeutics and diagnostic tools [34]. Recent research is therefore already focusing on the development of new compounds and antibodies directed against Aβ-pE(3) [21, 35–38] and the enzyme that catalyzes the formation of pyroglutamate Aβ, glutaminyl cyclase (QC) [39, 40]. Some of these compounds already show very promising results such as reduction of plaque load, degradation of Aβ-pE(3), inhibition of Aβ-pE(3) fibril formation, as well as rescue of learning and memory deficits in mice [21, 35, 37, 38].

### ptau Ser202/Thr205 in human tissue

Quantification of ptau Ser202/Thr205 immunoreactive area in several cortical regions revealed a late increase of ptau Ser202/Thr205 at Braak stage V/VI. Only in the hippocampus is an increase of ptau Ser202/Thr205 levels observed at Braak stage III/IV. These results are in agreement with our previous analyses using the same antibody on adjacent sections in similar brain regions of the same AD and control cases [41] suggesting that the used method of quantification results in only low deviations between independent experiments. Differences in ptau Ser202/Thr205 signal between studies might thus depend on slightly varying brain region of the same area. Our results strengthen previous findings that show a late increase in ptau Ser202/Thr205 levels [27]. The importance of ptau Ser202/Thr205 phosphorylation for AD was discussed in detail in our recent study about phosphorylation of different tau sites during progression of AD [41]. Inhibition of tau phosphorylation as AD treatment is thus a well-established target although no major breakthroughs could be established so far [42, 43].

### Animal models

Our results show strong and almost undistinguishable levels of Aβ-pE(3) in the cortex and hippocampus of APP_SL_ mice that significantly increased over age. First Aβ-pE(3) formation could be observed at 6–9 months of age and thus at the same age as observed by Mandler and colleagues [28] but much earlier as previously described by Frost and co-workers [44]. In 5xFAD mice, Aβ-pE(3) formation was even stronger and significantly increased at 6 months of age. This result is in agreement with results by Frost and colleagues [44], while Jawhar and co-workers observed an even earlier Aβ-pE(3) expression [45]. Although APP_SL_ and 5xFAD mice express only endogenous murine but no transgenic tau, a progressive increase of ptau Ser202/Thr205 could be observed in both models suggesting an influence of APP on posttranslational changes of tau [46]. As previously observed for Aβ-pE(3) [45], absolute levels of ptau Ser202/Thr205 were higher in 5xFAD compared to APP_SL_ mice. Cortical tau phosphorylation in 5xFAD mice was already described earlier [47] and can be assumed to be Aβ-induced, since both mouse models overexpress APP [47–49]. Increased tau phosphorylation in 5xFAD compared to APP_SL_ mice might be caused by transgenic mutated presenilin expression in 5xFAD mice, since presenilin has already been shown to induce tau phosphorylation [50, 51]. Correlation analyses of Aβ-pE(3) and ptau Ser202/Thr205 revealed strong correlations in the cortex and hippocampus of both mouse models. Strongest correlations could be observed in the cortex of APP_SL_ mice and in the hippocampus of 5xFAD mice, suggesting a different mode of action caused by the additional APP and presenilin mutations in 5xFAD mice.

### Correlation of Aβ-pE(3) and ptau Ser202/Thr205

Correlation analyses of Aβ-pE(3) and ptau Ser202/Thr205 revealed a highly significant relationship between these two proteins in all analyzed brain regions strengthening previous results [27] by using a different Aβ-pE(3) antibody, different human brain regions and analyzing different cortical regions in detail.

ptau Ser202/Thr205 phosphorylation seems highly associated to Aβ-pE(3), representing indeed a key link between tau and Aβ pathology. Due to this high dependency between ptau Ser202/Thr205 and Aβ-pE(3), it might be worth testing a combination therapy that targets both pathologies simultaneously. Efficacy analyses of new compounds against Aβ-pE(3) can be analyzed *in vivo* by using hQC, APP x hQC, TBA2.1 mice [52–54] or crossbreds with a common tau mouse model like TMHT [55] but also APP_SL_ and 5xFAD transgenic mice as shown here.

### Testing of Aβ-pE(3) antibodies

Two antibodies against Aβ-pE(3), msAβ-pE(3) and rbAβ-pE(3), were tested for specificity in late stage AD brain tissue as well as APP_SL_ and 5xFAD mouse brain. As most commercially available and also non-commercial Aβ-pE(3) antibodies described in the literature are from mice, and rabbit antibodies are sparse, we decided to compare the widely used Aβ-pE(3) antibody rbAβ-pE(3) from Synaptic Systems with the msAβ-pE(3) antibody from Vivoryon [25, 52, 56, 57]. The subtle differences of the two antibodies in labeling on human and mouse brain sections may be partly due to the mouse antibody being monoclonal and the rabbit antibody being polyclonal, since the polyclonal antibody may have multiple different binding sites at the immunogenic sequence, whereas mouse clone K17 has only one epitope. The qualitative labeling further shows that Aβ-pE(3) is highly abundant in senile plaques but represents only a part of total Aβ load as already shown by others [28]. We further tried to verify our results by performing Western blots of human brain tissue using the rbAβ-pE(3) and AT8 antibody as used for immunofluorescent quantification, but both antibodies did not result in proper signals on the human tissue. These results were unexpected, since both antibodies have been shown to result in good Western blot signals (S5 Fig and [58]). Western blots of ptau Ser202/Thr205 by ERP2402 antibody of the here analyzed human tissue were performed in our recent study supporting the here presented data [41].

## Conclusions

In summary, we endorse already published data about the correlation effects between Aβ-pE(3) and ptau Ser202/Thr205 and further strengthen them by analyzing several different cortical subregions in the human brain of Braak stage 0-VI using an Aβ-pE(3) antibody with validated specificity. By analyzing APP_SL_ and 5xFAD transgenic mice for correlation between Aβ-pE(3) and ptau Ser202/Thr205 we could further assess differences between the models caused by the different transgenes, evaluate similarities between the human and murine tissues and thus corroborate the value of these transgenic mouse models for future Aβ-pE(3) and ptau Ser202/Thr205 research.

## Supporting information

S1 Fig Immunofluorescent labeling of human cortical tissue of Braak stage V/VI with two different antibodies recognizing Aβ-pE(3), GFAP and labeling of cell nuclei with DAPI without antigen retrieval. Separate labeling of Aβ-pE(3) with antibodies of either mouse (msAβ-pE(3) a1, a2) or rabbit (rbAβ-pE(3) b1, b2) origin resulted in a strong and similar immunoreactive area. Both antibodies were additionally co-labeled with an antibody against GFAP (a1, a3, b1, b3) and cell nuclei were stained with DAPI (a-c, a4-c4). Double labeling with both Aβ-pE(3) antibodies (c1-c3) in contrast revealed a similar immunoreactive area of both antibodies. Missing overlap with recorded autofluorescence (a-c, a5-c5) highlights the general specificity of the Aβ-pE(3) and GFAP labelings or rather indicates unspecific objects. Magnification: 20x. AF: autofluorescence, Aβ-pE(3): pyroglutamate Aβ, DAPI: 4′,6-Diamidin-2-phenylindol, GFAP: glial fibrillary acidic protein.
(TIF)

S2 Fig Immunofluorescent labeling of Aβ-pE(3), GFAP, neurons and nuclei of cortical and hippocampal tissue of a 12 month old APP_SL_ and 5xFAD mouse. Labeling of Aβ-pE(3) using two different antibodies of either mouse (msAβ-pE(3), a, a2, b, b2) or rabbit (rbAβ-pE(3), a, a1, b, b2) origin resulted in a similar immunoreactive pattern. Also, separate incubation with both antibodies (rbAβ-pE(3) (c, c1, d, d1), msAβ-pE(3) (e, e1, f, f1) on two consecutive sections revealed a similar labeling of Aβ-pE(3). Visualization of GFAP was comparable in both sections (c, c2, d, d2, e, e2, f, f2). The diffuse (yellow arrow) as well as the densed core plaques (white arrow) were specifically labeled with both, the msAβ-pE(3) (g1, h1, i1, k1, g3, h3, i3, k3) and the rbAβ-pE(3) (g1, h1, i1, k1, g2, h2, i2, k2) antibody. However, using both antibodies together resulted in a slightly greater msAβ-pE(3)-positive immunoreactive area. Aβ-pE(3): pyroglutamate Aβ, DAPI: 4′,6-Diamidin-2-phenylindol, GFAP: glial fibrillary acidic protein, NeuN: neuronal nuclei.
(TIF)

S3 Fig Labeling of Aβ-pE(3), GFAP, cell as well as more specifically neuronal nuclei of a part of the cortex and the hippocampus of a 12 month old non-transgenic mouse. Labeling of Aβ-pE(3) using two different antibodies of either mouse (msAβ-pE(3), a, a2) or rabbit (rbAβ-pE(3), a, a1) origin resulted in no Aβ-pE(3) signal in ntg mice. Also labeling of Aβ-pE(3) using only the rbAβ-pE(3) antibody was negative (b, b1). b and b2 additionally show the labeling of GFAP for the visualization of astrocytes. Aβ-pE(3): pyroglutamate Aβ, DAPI: 4′,6-Diamidin-2-phenylindol, GFAP: glial fibrillary acidic protein, NeuN: neuronal nuclei.
(TIF)

S4 Fig Labeling of Aβ-pE(3), total Aβ (and APP, 6E10), nuclei and staining with Thioflavin S of the dentate gyrus of a 9 months old APP_SL_ (a-a4) and a 5xFAD (b-b4) mouse. Both, APP_SL_ and 5xFAD mice show labeling of total Aβ (and APP) with the 6E10 antibody (a, a1, a1´, b, b1, b1´), Aβ-pE(3) (a, a2, a2´, b, b2, b2´), staining with Thioflavin S (a, a3, a3´, b, b3, b3´) and cell nuclei, visualized by using DAPI (a, a4, a4´, b, b4, b4´). Merged images demonstrate the partial overlay of 6E10 labeled structures, Aβ-pE(3) and Thioflavin S stained objects (a, b). rbAβ-pE(3): Aβ-pE(3) of rabbit origin, ThioS: Thioflavin S. Sagittal sections.
(TIF)

S5 Fig Specificity test of antibodies msAβ-pE(3) and rbAβ-pE(3). 25 and 50 ng of Aβ-pE(3–40) (lane 1), Aβ-pE(3–42) (lane 2), Aβ 4–42 (lane 3) and Aβ 1–42 (lane 4) protein was blotted on a SDS PAGE gel, transferred to a nitrocellulose membrane and labeled with primary antibodies msAβ-pE(3) (**a**) and rbAβ-pE(3) (**b**). Afterwards, membranes were blotted with secondary antibodies against mouse or rabbit and visualized by luminescence.
(TIF)

## Abbreviations

AD: Alzheimer’s disease
ANOVA: Analysis of Variance
CiCtx: cingulate cortex
FrCtx: frontal cortex
NFT: neurofibrillary tangle
NT: neuropil threads
OcCtx: occipital cortex
ptau: phosphorylated tau
TeCtx: temporal cortex
TEntR: transentorhinal region
