# Supplementary figures and images for: Correlation of pyroglutamate amyloid β and ptau Ser202/Thr205 levels in Alzheimer’s disease and related murine models
Source: PLoS One. 2020 Jul 9;15(7):e0235543. doi: 10.1371/journal.pone.0235543 (PMC7347153; doi:10.1371/journal.pone.0235543)

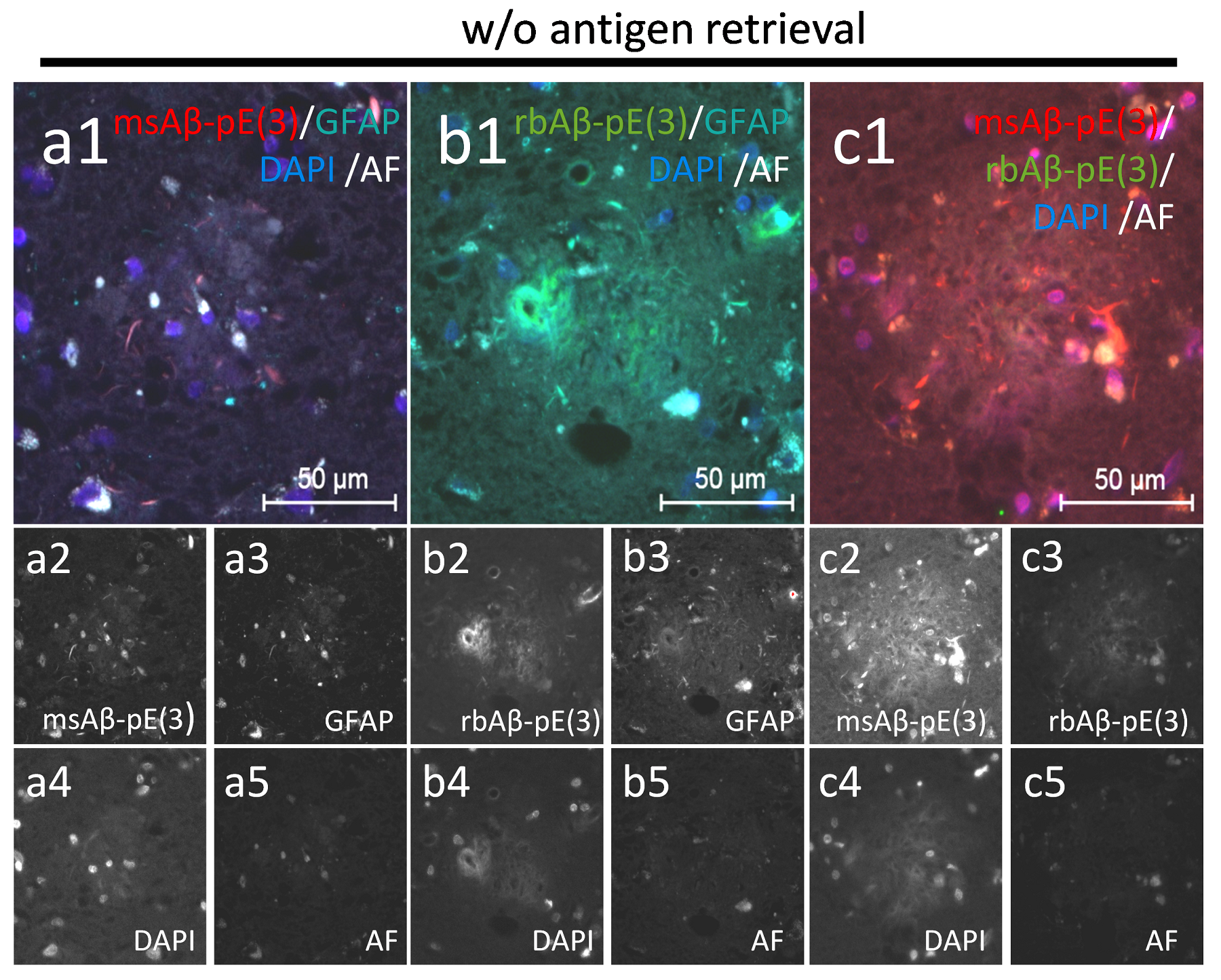

Supplement: S1 Fig — Separate labeling of Aβ-pE(3) with antibodies of either mouse (msAβ-pE(3) a1, a2) or rabbit (rbAβ-pE(3) b1, b2) origin resulted in a strong and similar immunoreactive area. Both antibodies were additionally co-labeled with an antibody against GFAP (a1, a3, b1, b3) and cell nuclei were stained with DAPI (a-c, a4-c4). Double labeling with both Aβ-pE(3) antibodies (c1-c3) in contrast revealed a similar immunoreactive area of both antibodies. Missing overlap with recorded autofluorescence (a-c, a5-c5) highlights the general specificity of the Aβ-pE(3) and GFAP labelings or rather indicates unspecific objects. Magnification: 20x. AF: autofluorescence, Aβ-pE(3): pyroglutamate Aβ, DAPI: 4′,6-Diamidin-2-phenylindol, GFAP: glial fibrillary acidic protein. (TIF) [file pone.0235543.s001.tif]

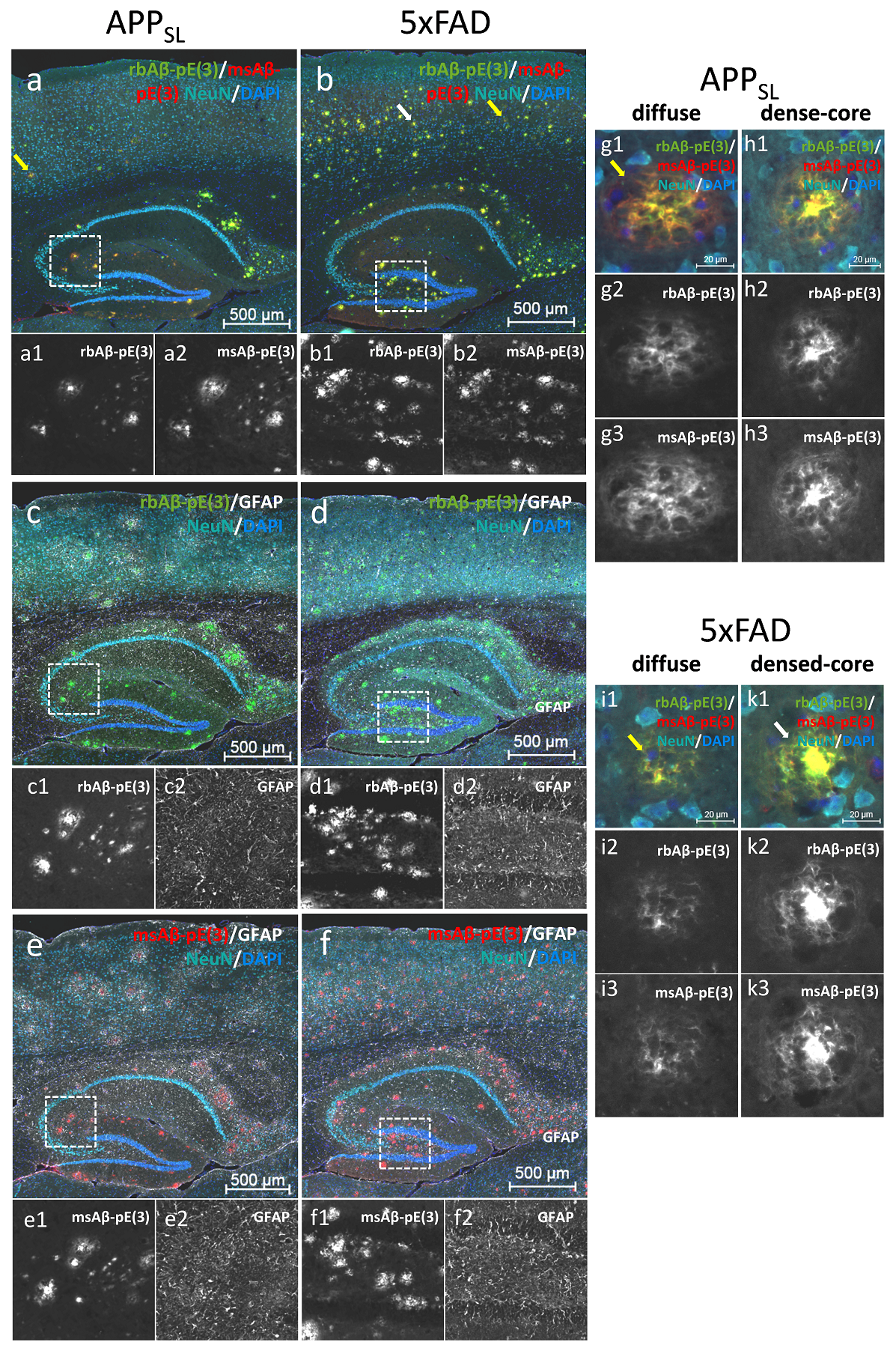

Supplement: S2 Fig — Labeling of Aβ-pE(3) using two different antibodies of either mouse (msAβ-pE(3), a, a2, b, b2) or rabbit (rbAβ-pE(3), a, a1, b, b2) origin resulted in a similar immunoreactive pattern. Also, separate incubation with both antibodies (rbAβ-pE(3) (c, c1, d, d1), msAβ-pE(3) (e, e1, f, f1) on two consecutive sections revealed a similar labeling of Aβ-pE(3). Visualization of GFAP was comparable in both sections (c, c2, d, d2, e, e2, f, f2). The diffuse (yellow arrow) as well as the densed core plaques (white arrow) were specifically labeled with both, the msAβ-pE(3) (g1, h1, i1, k1, g3, h3, i3, k3) and the rbAβ-pE(3) (g1, h1, i1, k1, g2, h2, i2, k2) antibody. However, using both antibodies together resulted in a slightly greater msAβ-pE(3)-positive immunoreactive area. Aβ-pE(3): pyroglutamate Aβ, DAPI: 4′,6-Diamidin-2-phenylindol, GFAP: glial fibrillary acidic protein, NeuN: neuronal nuclei. (TIF) [file pone.0235543.s002.tif]

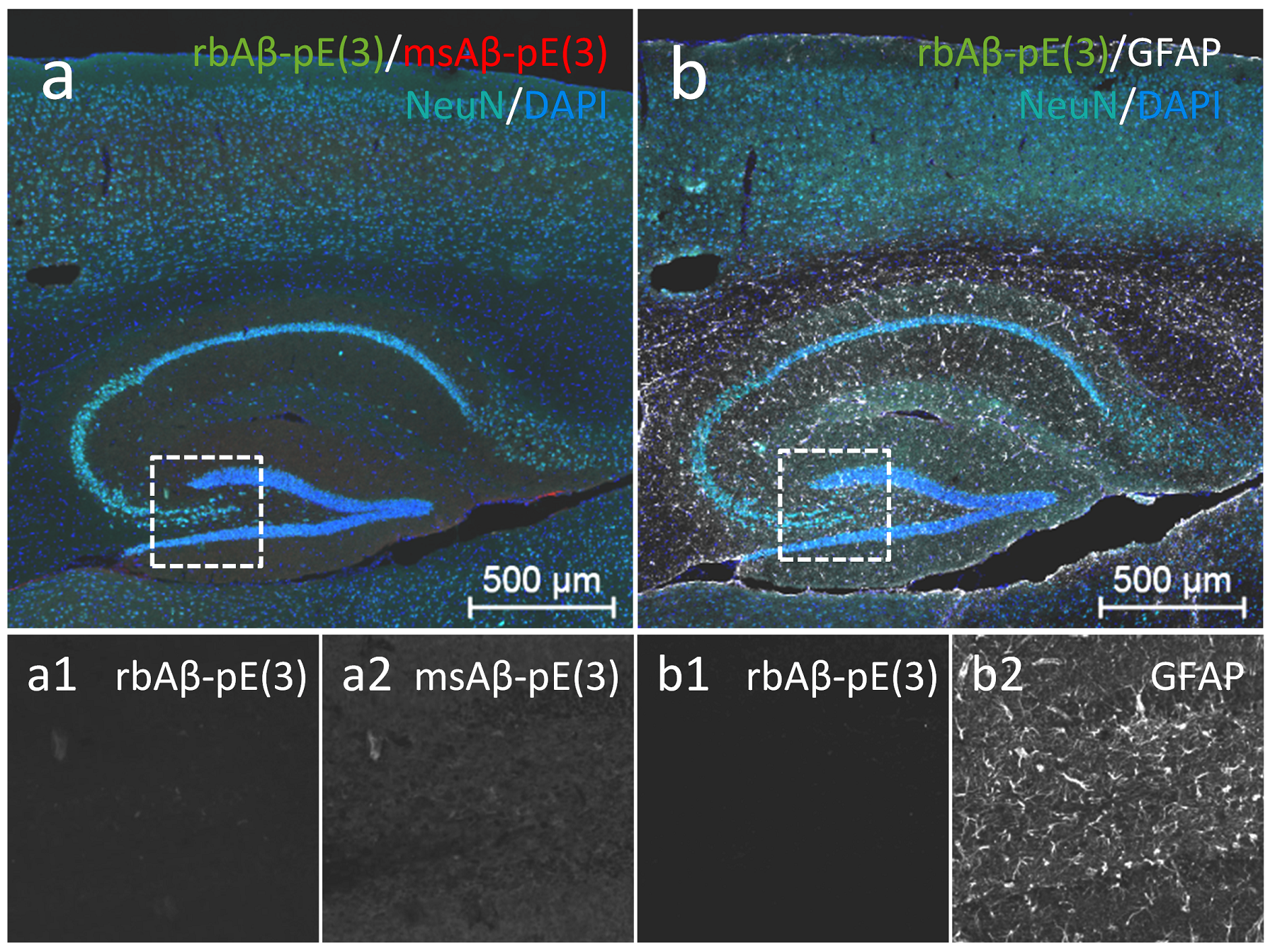

Supplement: S3 Fig — Labeling of Aβ-pE(3) using two different antibodies of either mouse (msAβ-pE(3), a, a2) or rabbit (rbAβ-pE(3), a, a1) origin resulted in no Aβ-pE(3) signal in ntg mice. Also labeling of Aβ-pE(3) using only the rbAβ-pE(3) antibody was negative (b, b1). b and b2 additionally show the labeling of GFAP for the visualization of astrocytes. Aβ-pE(3): pyroglutamate Aβ, DAPI: 4′,6-Diamidin-2-phenylindol, GFAP: glial fibrillary acidic protein, NeuN: neuronal nuclei. (TIF) [file pone.0235543.s003.tif]

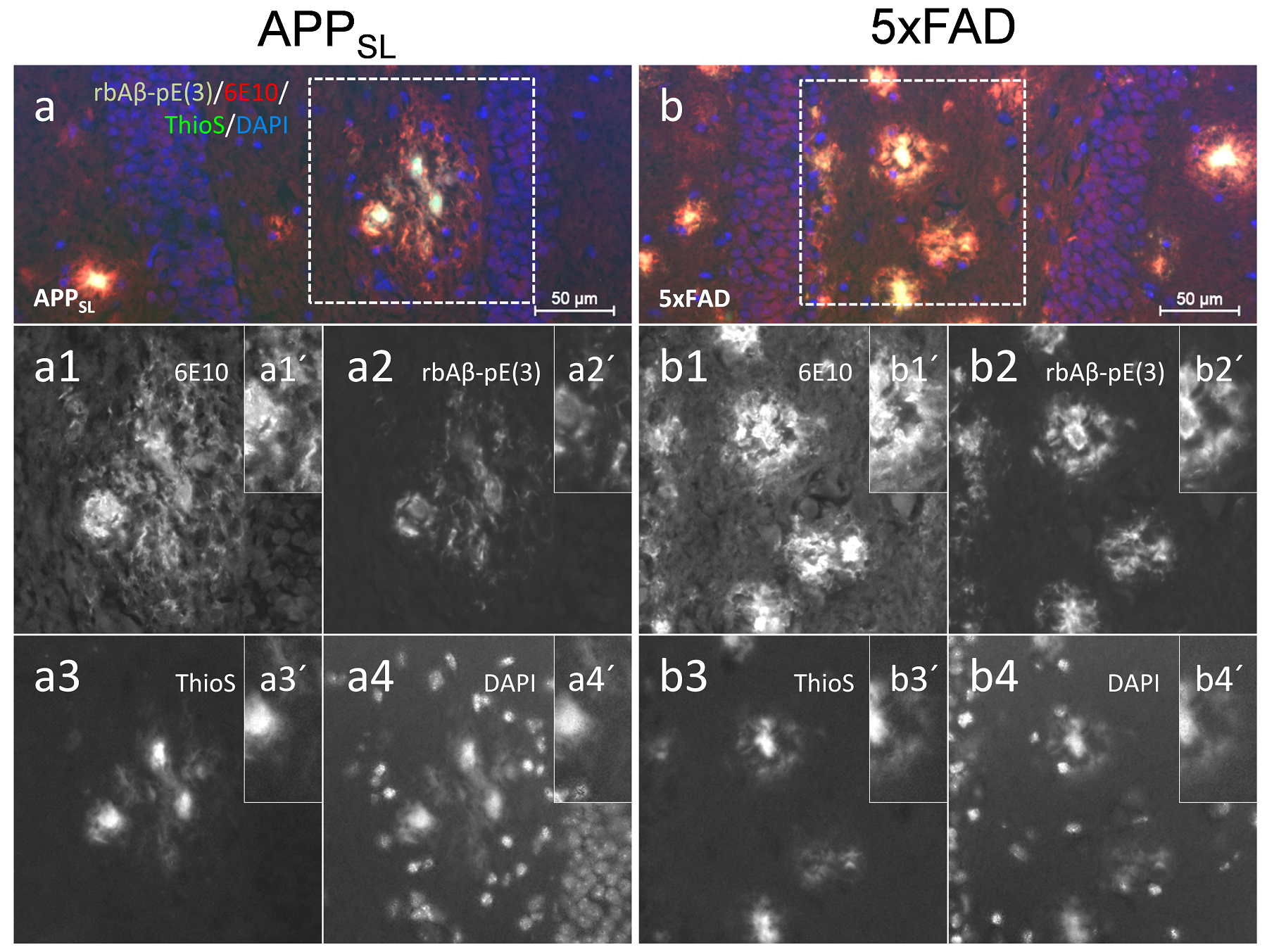

Supplement: S4 Fig — Both, APPSL and 5xFAD mice show labeling of total Aβ (and APP) with the 6E10 antibody (a, a1, a1´, b, b1, b1´), Aβ-pE(3) (a, a2, a2´, b, b2, b2´), staining with Thioflavin S (a, a3, a3´, b, b3, b3´) and cell nuclei, visualized by using DAPI (a, a4, a4´, b, b4, b4´). Merged images demonstrate the partial overlay of 6E10 labeled structures, Aβ-pE(3) and Thioflavin S stained objects (a, b). rbAβ-pE(3): Aβ-pE(3) of rabbit origin, ThioS: Thioflavin S. Sagittal sections. (TIF) [file pone.0235543.s004.tif]

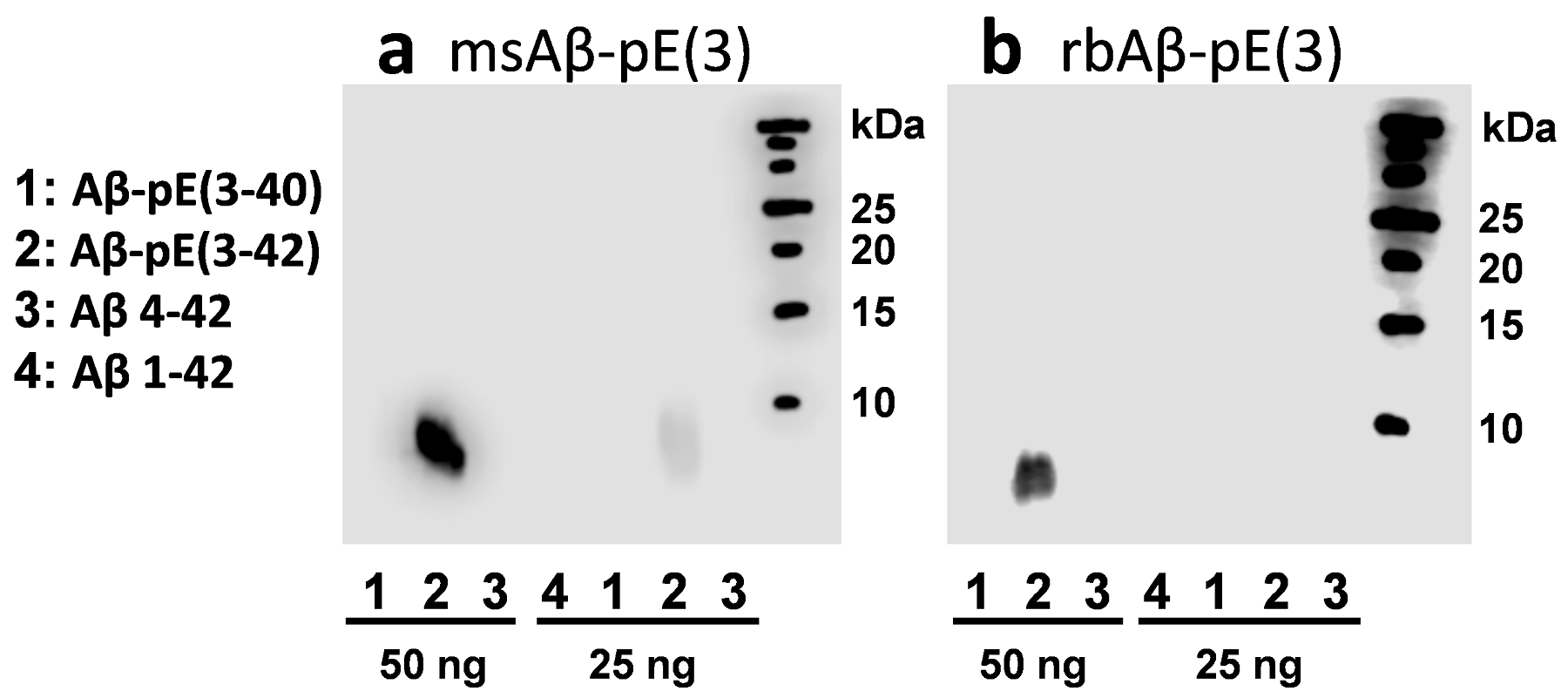

Supplement: S5 Fig — 25 and 50 ng of Aβ-pE(3–40) (lane 1), Aβ-pE(3–42) (lane 2), Aβ 4–42 (lane 3) and Aβ 1–42 (lane 4) protein was blotted on a SDS PAGE gel, transferred to a nitrocellulose membrane and labeled with primary antibodies msAβ-pE(3) (a) and rbAβ-pE(3) (b). Afterwards, membranes were blotted with secondary antibodies against mouse or rabbit and visualized by luminescence. (TIF) [file pone.0235543.s005.tif]
